# Analyzing ChatGPT adoption drivers with the TOEK framework

**DOI:** 10.1038/s41598-023-49710-0

**Published:** 2023-12-19

**Authors:** Hyeon Jo, Youngsok Bang

**Affiliations:** 1 Headquarters, HJ Institute of Technology and Management, 71 Jungdong-ro 39, Bucheon-si, Gyeonggi-do 14721 Republic of Korea; 2 School of Business, Yonsei University, 50 Yonsei-ro, Seodaemun-gu, Seoul, 03722 Republic of Korea

**Keywords:** Engineering, Mathematics and computing

## Abstract

With the rapid advancements in AI technology and its growing impact on various aspects of daily life, understanding the factors that influence users' adoption intention becomes essential. This study focuses on the determinants affecting the adoption intention of ChatGPT, an AI-driven language model, among university students. The research extends the Technology-Organization-Environment (TOE) framework by integrating the concept of knowledge application. A cross-sectional research design was employed, gathering data through a survey conducted to university students. Structural equation modeling was used to analyze the data, aimed at examining the relationships between key determinants influencing adoption intention. The findings of this research indicate that factors such as network quality, accessibility, and system responsiveness contribute to satisfaction. Furthermore, satisfaction, organizational culture, social influence, and knowledge application significantly affect adoption intention. These findings offer both theoretical and practical implications.

## Introduction

The rapid progression of artificial intelligence (AI) technologies has brought about substantial changes in various aspects of human life, including how we learn and communicate^1^. Lately, AI-powered language models like ChatGPT have garnered significant interest for their potential to reshape education, particularly within university environments^2^. ChatGPT, created by OpenAI, is an advanced AI language model capable of producing human-like text responses and assisting users in diverse tasks such as answering questions, offering explanations, and even creative writing^3^. Integrating ChatGPT into university students' learning experiences can considerably enhance their educational journey by delivering personalized, immediate support, encouraging critical thinking, and promoting knowledge application^4^. With a surge in its adoption, especially among university students, it becomes imperative to delve deeper into the nuances and intricacies that inform and influence its acceptance. Previous research has predominantly been skewed toward user experience, functionality, and technical prowess of such tools^5–9^. However, a holistic exploration that encompasses the trifecta of technological, organizational, and environmental factors remains largely untapped. Therefore, this study aims to bridge this existing gap, offering a comprehensive understanding of the determinants that shape ChatGPT's adoption intention among university students.

Existing research on technology adoption has primarily focused on the Technology-Organization-Environment (TOE) framework, which examines the technological, organizational, and environmental factors that account for the adoption of innovations^10–12^. The framework serves as a central theoretical lens for understanding the adoption and implementation of technological innovations within organizations^12^. This framework suggests that the adoption decision is influenced by three primary contextual domains: technological context (characteristics of the technology itself), organizational context (descriptive measures of the organization, such as size and scope), and environmental context (the arena in which an organization conducts its business, such as industry characteristics and regulatory environment)^12^.

ChatGPT represents an innovative technology that offers unique capabilities and the potential to transform educational experiences^13^. Rogers^14^ defines an innovation as a new idea, practice, or object that is perceived by an individual or group as new. Due to ChatGPT's advanced natural language processing capabilities that allow it to generate text responses similar to those produced by humans and assist users with various tasks^15,16^, the TOE framework is an appropriate tool to explore the factors influencing ChatGPT adoption intentions.

Due to the sudden surge in users, ChatGPT has shown a variety of technical flaws. ChatGPT occasionally showed network errors^17^. This demonstrated the importance of network quality, which directly impacts the system's performance, reliability, and speed^18^. Some users have encountered difficulties accessing chat history^19^. Accessibility is another critical technological context factor that influences ChatGPT adoption. Ensuring that ChatGPT is readily accessible to university students can significantly increase its adoption potential. ChatGPT's response is often delayed due to a login loop or internal server error^20^. A system that provides accurate and timely responses to user queries is more likely to be adopted and used by university students^21^.

Organizational culture significantly influences the attitudes, beliefs, and behaviors of individuals within an organization^22^. A strong organizational culture that values technological advancements and supports their use can create a positive atmosphere, ultimately leading to a higher adoption intention among university students.

Social influence may be another essential factor that can significantly impact the intention of ChatGPT adoption among university students^23,24^. By leveraging social influence, universities can create a supportive environment that fosters the adoption of ChatGPT through platforms that enable students to share their experiences and success stories, or even establish ambassador programs to promote the tool among their peers.

In addition, college students can apply the knowledge gained from ChatGPT to personal tasks, teamwork, and continued learning. Knowledge application could be an additional determinant of adoption intention. The decision to incorporate knowledge application as a determinant in understanding college students' intention to adopt ChatGPT is based on the Technology Acceptance Model (TAM), which was introduced by Davis^25^. According to TAM, the adoption of technology is influenced by its perceived usefulness. Perceived usefulness refers to an individual's belief that the utilization of a specific technology can positively impact their task performance. In the case of ChatGPT adoption among university students, knowledge application can be viewed as an aspect of perceived usefulness. Students who regard ChatGPT as a beneficial resource to apply their learned knowledge in various academic pursuits, such as decision-making, problem solving, and teamwork, are more inclined to adopt the technology. By adding knowledge application to the research model, the study recognizes the importance of perceived usefulness in shaping the adoption intentions of university students, providing a more comprehensive insight into the factors that affect their adoption choices. In the context of ChatGPT adoption among university students, knowledge application can be regarded as a dimension of perceived usefulness. By incorporating knowledge application into the research model, the study acknowledges that students who perceive ChatGPT as a valuable tool to apply their knowledge in various academic tasks are more likely to adopt the technology.

The selection of the TOE framework was strategic and rooted in its contextual relevance to our study. Unlike the widely recognized TAM, which is predominantly centered on perceived utility and ease of operation, the TOE framework offers a more encompassing perspective. TAM, while invaluable, might not thoroughly address the expansive organizational, technological, and environmental variables imperative to understand the adoption of ChatGPT among university students. On the other hand, the TOE framework holistically interweaves these dimensions, making it more suitable for an educational setting. In the technological context, we employ attributes like network quality, accessibility, and system response of ChatGPT. In the organizational context, we reflect the influence of organizational culture, which in this study refers to educational settings and the predominant attitudes and behaviors within universities. In the environmental context, we consider social influence, which refers to the broader impact of the academic community on students. Thus, each component of our research model can be distinctly mapped back to the foundational pillars of the TOE framework, showcasing a seamless alignment. Furthermore, our initiative to infuse TOE with the element of knowledge application underscores our effort to tailor the model to the distinct intricacies of assimilating AI-driven language models within tertiary education. To sum it up, our conviction was that an augmented TOE framework would provide a more rounded viewpoint to discern the factors that influence ChatGPT adoption in an academic setting.

The purpose of this study is to investigate the determinants of the intention to adopt ChatGPT among university students by extending the TOE framework to include a knowledge context. Specifically, the research aims to examine the relationships between network quality, accessibility, system response, satisfaction, organizational culture, social influence, knowledge application, and adoption intention. By incorporating the knowledge context into the TOE framework, the research contributes to a more comprehensive understanding of the factors influencing the adoption of AI-driven language models in university settings.

Our study stands distinct in its contributions to the existing literature in several significant ways as follows. First, while the TOEK framework has been extensively applied in exploring the adoption of various technological tools, its application to ChatGPT, an AI-driven communication tool, remains unprecedented. Therefore, this study fills this framework-application gap, enriching the literature on technology adoption models. Second, most prior studies have either focused on individual determinants or specific sectors when exploring the adoption of AI-driven platforms^26–28^. Our research offers a comprehensive exploration, incorporating technology, organization, environmental, and knowledge factors together, thus providing a holistic perspective on adoption intention. Third, previous research has predominantly targeted professionals or general users^6,29–31^. By narrowing our focus to university students, our study sheds light on a crucial demographic, enhancing the granularity of insights in the literature. Finally, while many studies have highlighted technological and organizational determinants, the intersection of knowledge variables with adoption intention has been less explored. Our inclusion of knowledge application provides a more sophisticated understanding of adoption behaviors, further enriching the literature.

## Background and related work

The adoption of AI chatbots and language models like ChatGPT has gained considerable attention in recent years, particularly in the context of education and learning^26,28,32–34^. These AI-driven tools have been recognized for their potential to revolutionize learning experiences by providing personalized and real-time support, fostering critical thinking, and facilitating knowledge application^2,4^.

Research on AI artifact adoption has often employed theoretical frameworks, such as the TAM^25^ and the unified theory of acceptance and use of technology (UTAUT)^79^, to investigate the factors influencing the adoption intention of users^26,35,36^. In addition to the technological factors for the introduction of ChatGPT, it can be explained by organizational culture or social impact. Universities can encourage college students to study and at the same time provide room for cheating. The culture of universities can promote or limit students' use of ChatGPT. The rapid development of ChatGPT was made by social influences such as the mention of online media and acquaintances. The more people around you mention ChatGPT to college students, the more they try to introduce it. Therefore, this study explains the intention of adoption by applying the TOE framework. The TOE framework is a widely adopted theoretical model that has been extensively used to investigate the determinants of information system adoption, including chatbots^37–39^. The TOE framework posits that the adoption of new technologies is influenced by a combination of technological, organizational, and environmental factors.

### Technology and ChatGPT

The technological context in the TOE framework refers to the characteristics of the technology itself and how they impact its adoption. Several studies have identified factors such as network quality^40,41^, accessibility^42^, and system response^43^ as crucial determinants of technology adoption. A high-quality network ensures seamless access to ITs and enhances user satisfaction, ultimately affecting adoption intention^44^. Ease of access, integration with existing platforms, and user-friendly interfaces are vital in determining user satisfaction and adoption intention^45–47^. The system response of the AI chatbot drives user experience and satisfaction^26,48^. ChatGPT has been problematic in terms of network quality^17^, accessibility^19^, and system responsiveness^20^ among many technical factors since its launch. Therefore, this study confirms that these three factors are correlated with satisfaction with ChatGPT.

### Organization and ChatGPT

Organizational context, another key component of the TOE framework, encompasses factors such as organizational size, structure, culture, and resources^49,50^. Organizational culture includes fostering a technology-friendly environment, promoting faculty and staff training, and integrating AI-driven tools into the curriculum^51,52^. Organizational culture has been found to impact technology adoption in various settings, including educational institutions^32–34^.

In our study, the incorporation of organizational culture within the organizational context is pivotal to understanding the adoption of ChatGPT among university students. This construct, in our context, includes the university's support for using ChatGPT, initiatives like incentive programs to promote its use, and the positive influence of university policies on ChatGPT adoption^53^. The stance of a university towards innovative technologies such as ChatGPT critically shapes student attitudes and behaviors towards adoption. A supportive university environment, demonstrated through policies and incentives, fosters a conducive atmosphere for exploring and integrating advanced tools like ChatGPT. This supportive culture not only encourages the acceptance and use of these technologies in academic routines but also influences broader trends in institutional technology adoption.

### Environment and ChatGPT

The environmental context of the TOE framework considers factors external to the organization that can impact technology adoption. This study aligns social influence with the environmental context of the TOE framework because it encapsulates the external pressures or influences that impact an individual's decision to adopt new technology. Social influence in our study refers to the impact of peers, user communities, and the broader social environment on university students’ decisions to adopt ChatGPT^54^. Social influence may act as a powerful environmental factor that shapes user behavior and adoption intention towards innovative technologies like ChatGPT. According to the social influence theory^54^, people tend to conform to the opinions and behaviors of their peers, superiors, and other influential individuals or groups. In the context of ChatGPT, positive word-of-mouth, peer recommendations, and testimonials can encourage students to adopt the tool^55^. Social influence has emerged as a significant environmental factor, with numerous studies demonstrating the importance of peer recommendations, positive word-of-mouth, and testimonials in encouraging users to adopt chatbots^23,24^.

### Knowledge and ChatGPT

Incorporating knowledge context into the TOE model while exploring college students' intention to adopt ChatGPT offers a more comprehensive understanding of the factors driving technology adoption in educational settings. The knowledge context, which focuses on the practical application of acquired information, complements the technological, organizational, and environmental factors in the TOE model by emphasizing the role of perceived usefulness in technology adoption. For college students, the ability to apply the knowledge gained from Chatbots in various academic tasks is crucial in determining the technology's adoption^56^. By adding the knowledge context to the TOE model, the study acknowledges that students who perceive ChatGPT as a valuable tool for applying their knowledge are more likely to adopt the technology.

In our research, the inclusion of knowledge application within the knowledge context is crucial for a comprehensive understanding of ChatGPT's adoption among university students. This construct is measured through three primary dimensions: ease of access to various knowledge types, the integration of different knowledge forms, and the enhancement of university learning^57^. These dimensions reflect the practical utility of ChatGPT as a tool for academic advancement. By facilitating instant access to diverse knowledge, enabling the integration of various information sources, and aiding in mastering academic content, ChatGPT serves as a critical resource for students in their educational pursuits. The inclusion of these measures aligns with Al-Sharafi et al.^57^, who emphasize the significance of knowledge application in technology adoption within academic settings. By incorporating this context, we acknowledge the evolving educational needs of students and how AI-driven tools like ChatGPT can meet these needs by providing tailored, interactive learning experiences.

## Research model

**Figure 1:**
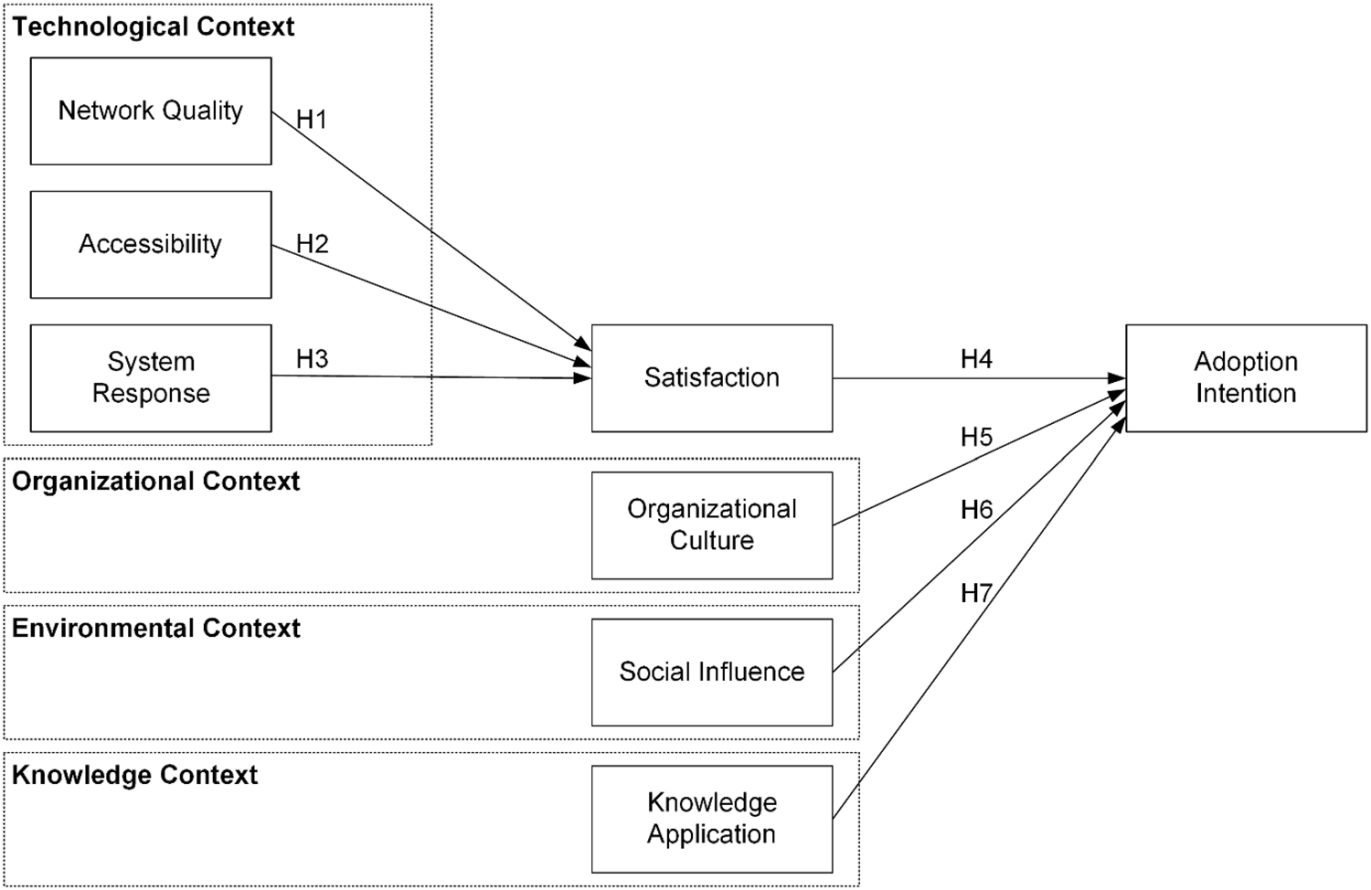
The conceptual framework.

The research model employed in Fig. 1 is an augmented version of the TOE framework, which incorporates a knowledge context to better understand the determinants of the intention to adopt ChatGPT among university students. The technological factors considered in the model are network quality, accessibility, and system response, which are hypothesized to have significant relationships with user satisfaction. Moreover, this study posits that organizational culture, social influence, and knowledge application have a correlation with adoption intention.

In choosing the constructs for our research, our primary focus was on the pressing technical issues that arose during the initial phase of ChatGPT adoption. At that time, there were predominant concerns about network quality, accessibility, and system response. We anchored our selection process in the theoretical foundation provided by DeLone and McLean^18^'s Information System Success Model, which underscores system quality, information quality, and service quality as primary antecedents of information system success. However, the predominant issues reported with ChatGPT at the time were less information quality and more system functionality, accessibility, and network stability. Moreover, unlike enterprise information systems that are often facilitated by external experts for individual users, ChatGPT lacked a comparable service quality environment; hence, we chose to exclude service quality as a construct. The sudden spike in ChatGPT users exposed various technical glitches, from occasional network errors^17^, indicating the importance of network quality in performance, reliability, and speed^18^, to accessibility challenges in retrieving chat histories^19^, and delays in system responses due to internal server errors^20^. Such accurate and timely system responsiveness is paramount for adoption, especially among university students^21^.

Moreover, while the TOE framework typically centers on elucidating enterprise information systems within industrial contexts, we intentionally excluded variables exclusive to enterprise systems. For instance, concepts like top management support are not applicable to a university student's decision to adopt ChatGPT due to the absence of supervisors or managers in academic contexts. Similarly, constructs such as firm size were deemed irrelevant as the size of an institution does not necessarily influence individual adoption decisions. Other factors, including competitive pressure, business partnerships, and government support, either were not pertinent to the South Korean context or did not align with the academic milieu of our study.

### Network quality

The relationship between network quality and satisfaction is well-established in the information systems literature. Network quality refers to the performance of the underlying infrastructure that supports a technology or service, encompassing factors such as reliability, speed, and latency^58,59^. A high-quality network is essential to ensure smooth interactions with technology like ChatGPT, as it directly affects the user experience. Research has shown that network quality is a significant determinant of user satisfaction in various contexts, such as e-commerce^60^ and mobile services^61,62^. Better network quality leads to reduced response times, fewer errors, and an overall enhanced user experience, which in turn contributes to increased satisfaction^63^. Therefore, this paper suggests the following:

#### H1

Network quality of ChatGPT has a positive correlation with satisfaction.

### Accessibility

Accessibility is an essential factor that refers to the ease with which users can access, download, and retrieve information using a specific technology^64^. It plays a crucial role in shaping user satisfaction; the more accessible technology is, the more likely users are to find it valuable and beneficial. A high level of accessibility in ChatGPT can lead to greater user satisfaction among university students. Previous research has shown that accessibility is a critical determinant of user satisfaction in various contexts, such as e-learning^43^ and mobile services^65^. When users can easily access, download, and retrieve information using ChatGPT, they are more likely to have a satisfying experience^66,67^. Consequently, enhancing the accessibility of ChatGPT may contribute to increased user satisfaction. Thus, this study proposes the following:

#### H2

Accessibility of ChatGPT has a positive correlation with satisfaction.

### System response

System response refers to the speed, accuracy, and reliability with which a technology responds to user input and delivers the desired outcome^43^. In the context of ChatGPT, system response encompasses the tool's ability to provide relevant, accurate, and timely responses to users' inquiries or requests. High-quality system response in ChatGPT can significantly impact user satisfaction among university students. Recent studies have highlighted the importance of system response in determining user satisfaction in various technology contexts, such as e-learning^68^ and AI artifact^69^. Users are more likely to be satisfied with ChatGPT when it provides prompt, accurate, and reliable responses to their inquiries, which, in turn, can lead to continued use and positive word-of-mouth. Thus, enhancing the system response of ChatGPT may contribute to increased user satisfaction. Thus, this paper suggests the following:

#### H3

System response of ChatGPT has a positive correlation with satisfaction.

### Satisfaction

Satisfaction, in the context of technology adoption, refers to the extent to which users are pleased with their experience using a product or service^70^. The relationship between satisfaction and adoption intention has been widely studied in various domains^71,72^. A positive link between satisfaction and the intention to adopt technology has been consistently reported in the literature on AI^26,73–75^. In the context of ChatGPT, when users are satisfied with their experience, they are more likely to develop an intention to adopt the technology. Therefore, understanding the role of satisfaction in shaping adoption intention is crucial for the development and marketing of AI-driven tools like ChatGPT. This study hypothesizes the following:

#### H4

Satisfaction with ChatGPT has a positive correlation with adoption intention.

### Organizational culture

Organizational culture is a set of shared values, beliefs, and practices within an organization that influence the behavior of its members^22,53^. In the context of universities, organizational culture can play a significant role in shaping the adoption of new technologies, such as ChatGPT. A supportive organizational culture can foster innovation and facilitate the adoption of new technologies within universities^76^. For example, a university with a culture that encourages collaboration, openness to change, and continuous learning is more likely to have its members willing to adopt AI-driven tools like ChatGPT^77^. In contrast, a rigid organizational culture may hinder technology adoption, as members can resist change and feel threatened by new tools^78^. Therefore, understanding the impact of organizational culture on the intention to adopt ChatGPT is essential for its successful integration within universities. Thus, this research proposes the following:

#### H5

Organizational culture has a positive correlation with adoption intention.

### Social influence

Social influence refers to the phenomenon in which an individual's beliefs, attitudes, and actions are shaped or altered by the presence or actions of other people^54^. Social influence can have a substantial impact on the adoption of technology, as individuals may be more likely to embrace new tools if they see others doing so successfully^79^. Previous studies have found that social influence can have a positive impact on the adoption of various technologies, including e-learning platforms^80^ and mobile applications^81^. In the context of ChatGPT, social influence can shape adoption intentions through peer recommendations, perceived norms, and observations of successful usage among colleagues^82^. If university students perceive that their peers and faculty members have positive experiences with ChatGPT, they are more likely to develop a favorable attitude toward the tool and adopt it themselves^83,84^. Thus, this study proposes the following:

#### H6

Social influence has a positive correlation with adoption intention.

### Knowledge application

Knowledge application refers to the process of utilizing acquired knowledge to make decisions, generate new ideas, or solve problems^85^. Recent studies have demonstrated the importance of knowledge application in the adoption of various technologies, such as e-learning platforms^86^ and decision support systems^87^. In the context of ChatGPT, the ability to apply knowledge effectively may facilitate university students' learning, problem-solving, and decision-making, consequently increasing their intention to adopt the tool^88^. For instance, ChatGPT's natural language processing capabilities can help students synthesize information, generate summaries, and create new ideas^89^. Thus, this study proposes the following:

#### H7

Knowledge application has a positive correlation with adoption intention.

In the research model, gender, age, education, and income are considered control variables due to their potential influence on the adoption intention of ChatGPT among university students.

## Methodology

This research was performed in accordance with the Declaration of Helsinki.

### Measurement instrument

To effectively examine the factors influencing the adoption intention of ChatGPT among university students, an appropriate research instrument was developed to measure the constructs in the model, which include network quality, accessibility, system response, satisfaction, organizational culture, social influence, and knowledge application. A detailed review of previous studies on technology adoption was conducted, focusing on TOE framework applications and AI chatbot adoption. Established scales from prior research were adapted to fit the context of ChatGPT adoption among university students. The items were modified to ensure that they accurately reflected the context of ChatGPT use in educational settings. The adapted items were then reviewed by a panel of experts, including researchers in technology adoption and university faculty experienced in using AI-driven tools. The panel provided feedback on the content validity of the items, and revisions were made accordingly to ensure that the items adequately captured the constructs of interest. A pilot test was conducted with 20 participants to assess the reliability and validity of the adapted instrument. A sample of university students, who had experience with ChatGPT, was recruited to participate in the pilot study. Participants were asked to respond to the items using a 7-point Likert scale, ranging from 1 (strongly disagree) to 7 (strongly agree). The refined instrument, consisting of the final set of items for each construct, was used in the main study to assess the factors influencing the adoption intention of ChatGPT among university students. Table A1 details the constructs and each item.

### Subject and data collection

The study targeted university students as subjects to investigate the factors that influence the intention of ChatGPT adoption. To achieve a comprehensive representation, we distributed our survey to students across diverse disciplines and universities located in various regions of the country. A convenience sampling technique was used to recruit participants for this study. Distribution channels included direct referrals from several professors, postings on university student communities, and platforms frequented by ChatGPT users. Our participants spanned the entire academic spectrum, from undergraduate students to those in master's and doctoral programs, and even included post-doctoral researchers. To ensure the relevance of our survey to active users, a screening question was incorporated at the start of our survey to determine whether the respondent was currently using ChatGPT. Participation in the study was voluntary, and the respondents were assured of their confidentiality and anonymity. An online survey was conducted using the refined instrument developed in the previous section. A brief introduction to ChatGPT, along with an explanation of its potential applications in educational settings, was provided to the respondents. The data collection period lasted for three weeks from late March to April 2023.

Addressing response bias was crucial to ensure the credibility and reliability of our research findings. To manage this bias, we took several measures. First, the questionnaire included a mix of positive and negative items. This was designed to prevent consistent response patterns and to ensure that respondents actively engaged with each question. Second, as previously mentioned, we conducted a pilot test before the main survey to collect feedback on the clarity and structure of the question. This helped reduce potential misunderstandings that could lead to biased responses. Third, respondents were ensured of the confidentiality of their answers, promoting genuine and honest feedback. Lastly, before finalizing our data for analysis, we performed an initial data screening to identify and eliminate potential outliers or patterns indicative of response bias. We inspected the dataset for missing values, duplicate entries, or inconsistent responses. Incomplete or inconsistent responses were excluded, resulting in 233 valid responses for further analysis.

The determination of the sample size in structural equation modeling (SEM) often considers several factors, including the complexity of the model, the desired statistical power level, the anticipated effect size, and the probability level. While larger samples are generally preferred, it is also crucial to balance feasibility with the requirements of the research. According to the a-priori sample size calculator for SEM^90^, with an anticipated effect size of 0.1, desired power level of 0.8, eight latent variables, twenty-four observed variables, and a probability level of 0.05, the minimum recommended sample size for the model structure is 200. The sample size for this study was 233, which met the criteria.

Table 1 shows the demographic profile of the respondents. The majority of the participants were female (58.4%), while males accounted for 41.6% of the sample. In terms of age, 43.3% of the respondents were 20 years old or younger, 8.6% were 21, 10.3% were 22, and 37.8% were 23 years old or older. This distribution reflects a diverse representation of university students in terms of gender and age, allowing a comprehensive understanding of the factors influencing the intention to adopt ChatGPT.

**Table 1:** Profile of the respondents.

Demographics	Item	Subjects (N = 233)	
		Frequency	Percentage (%)
Gender	Male	97	41.6
	Female	136	58.4
Age	20 or younger	101	43.3
	21	20	8.6
	22	24	10.3
	23 or older	88	37.8
Education	Middle school	1	0.4
	High school	168	72.1
	Bachelor’s degree	53	22.7
	Master’s degree	7	3.0
	Doctor’s degree	4	1.7
Annual income (in million KRW)	Less than 10	205	88.0
	10–30	17	7.3
	30–50	9	3.9
	More than 50	2	0.9

### Ethical approval

This study was approved by an institutional review board of HJ Institute of Technology and Management.

### Consent to participate

Consent to participate was obtained from all individual participants included in the study.

### Informed consent

Informed consent was obtained from all individual participants included in the study.

## Analysis and results

To analyze the data and test the research hypotheses, this study employed partial least squares SEM (PLS-SEM) using SmartPLS 3.3.9 software. PLS-SEM was chosen because of its ability to handle complex models with multiple constructs and small to moderate sample sizes^91^. The analysis process was conducted in two stages: the assessment of the measurement model and the assessment of the structural model.

### Common method bias (CMB)

CMB refers to the potential for systematic error in the measurement of variables due to the use of a single method for data collection, such as self-report questionnaires^92^. CMB can lead to inflated relationships among variables, which can result in misleading or spurious findings. To address the potential issue of CMB in this study, several procedural and statistical remedies were applied.

The procedure remedies are as follows. Respondents were assured that their responses would remain anonymous and confidential, reducing the likelihood of social desirability bias and encouraging honest responses^92^. The data collection process was designed to minimize the potential for CMB by administering the survey in three separate sessions, with a one-week time gap between sessions. This temporal separation reduced the likelihood that respondents recall their previous responses and the potential for bias from the common method.

This study also conducted statistical remedies. First, a principal component factor analysis was conducted on all items to assess the presence of a single dominant factor that could account for the majority of the variance. The results revealed that no single factor accounted for a majority of the variance, with the first factor explaining only 36.871% of the total variance, which is below the 50% threshold suggested by^92^. This result indicates that common method bias is not a major concern in the data. Second, a marker variable, not related to the study constructs, was included in the questionnaire to assess the potential influence of common method bias. The correlations between the marker variable and the main study variables were examined. The low and non-significant correlations between the marker variable and the main study variables suggest that common method bias is not a significant issue in the data^93^. Last, in this study, we used variance inflation factors (VIF) as recommended in previous studies^94^. A VIF greater than 3.3 is considered a potential indicator of CMB. As shown in Table 2, our analysis found that the VIFs for all constructs were below the threshold. Therefore, this research concluded that CMB was unlikely to be a significant issue that could influence the results.

**Table 2:** VIF.

Variable 1	Variable 2	VIF
Network quality	Satisfaction	1.646
Accessibility	Satisfaction	1.131
System response	Satisfaction	1.643
Satisfaction	Adoption intention	1.771
Organizational culture	Adoption intention	1.140
Social influence	Adoption intention	1.478
Knowledge application	Adoption intention	1.833
Gender	Adoption intention	1.013
Age	Adoption intention	2.807
Education	Adoption intention	2.352
Income	Adoption intention	2.182

### Measurement model assessment

The measurement model was evaluated to ensure the reliability and validity of the constructs. This included examining the following criteria: First, all factor loadings were above the recommended threshold of 0.7^91^, indicating satisfactory indicator reliability. Composite reliability (CR) values were calculated for each construct. All CR values exceeded the suggested cut-off point of 0.7^91^, confirming the reliability of the constructs. The average variance extracted (AVE) was assessed for each construct. All AVE values were above the recommended value of 0.5^95^, indicating adequate convergent validity. Table 3 shows the results measurement model assessment.

**Table 3:** Test results of reliability and validity.

Construct	Items	Mean	St. Dev	Factor loading	Cronbach's alpha	CR	AVE
Network quality	NEQ1	4.828	1.363	0.901	0.897	0.936	0.829
	NEQ2	4.798	1.329	0.896			
	NEQ3	4.700	1.369	0.933			
Accessibility	ACS1	5.369	1.288	0.826	0.732	0.846	0.646
	ACS2	5.270	1.355	0.824			
	ACS3	5.039	1.385	0.761			
System response	SRP1	4.918	1.383	0.901	0.839	0.902	0.755
	SRP2	4.605	1.408	0.812			
	SRP3	4.987	1.227	0.891			
Satisfaction	SAT1	5.000	1.236	0.934	0.925	0.952	0.869
	SAT2	4.918	1.276	0.951			
	SAT3	4.906	1.277	0.912			
Organization culture	OGC1	4.094	1.514	0.866	0.870	0.920	0.794
	OGC2	3.794	1.488	0.919			
	OGC3	3.987	1.397	0.887			
Social influence	SOI1	5.365	1.165	0.919	0.920	0.949	0.862
	SOI2	5.155	1.281	0.927			
	SOI3	5.326	1.192	0.939			
Knowledge application	KAP1	5.245	1.248	0.876	0.821	0.893	0.737
	KAP2	5.064	1.233	0.884			
	KAP3	4.652	1.419	0.813			
Adoption intention	ADI1	5.073	1.494	0.920	0.885	0.929	0.813
	ADI2	4.356	1.649	0.863			
	ADI3	5.039	1.442	0.921			

The Fornell-Larcker criterion and cross-loadings were examined to assess discriminant validity. The square root of AVE for each construct was higher than its correlations with other constructs, and the cross-loadings of the indicators were higher within their respective constructs than with other constructs, supporting the discriminant validity of the measurement model. Table 4 shows the correlation matrix and the discriminant assessment.

**Table 4:** Correlation matrix and discriminant assessment.

Construct	1	2	3	4	5	6	7	8
1. Network	0.910							
2. Accessibility	0.307	0.804						
3. System response	0.614	0.305	0.869					
4. Satisfaction	0.421	0.517	0.445	0.932				
5. Organizational culture	0.141	0.131	0.193	0.280	0.891			
6. Social influence	0.258	0.316	0.345	0.450	0.245	0.929		
7. Knowledge application	0.390	0.395	0.376	0.619	0.278	0.466	0.858	
8. Adoption intention	0.262	0.343	0.290	0.537	0.374	0.699	0.548	0.902

Table 5 presents the Heterotrait-Monotrait (HTMT) ratio of correlations among the study constructs. The HTMT values range from 0.163 to 0.769, suggesting adequate discriminant validity among the constructs. It can be observed that the highest value (0.769) occurs between social influence and adoption intention, indicating a strong relationship between these two constructs. conversely, the lowest value (0.163) is observed between organizational culture and network, suggesting a weak relationship between these constructs. The remaining HTMT values fall below the recommended threshold of 0.85 (Henseler et al., 2015), further confirming the discriminant validity of the constructs in the research model.

**Table 5:** HTMT ratio.

Constructs	1	2	3	4	5	6	7	8
1. Network								
2. Accessibility	0.378							
3. System response	0.689	0.373						
4. Satisfaction	0.460	0.613	0.500					
5. Organizational culture	0.163	0.172	0.222	0.313				
6. Social influence	0.286	0.369	0.376	0.485	0.270			
7. Knowledge application	0.452	0.494	0.441	0.709	0.328	0.530		
8. Adoption intention	0.298	0.397	0.327	0.590	0.428	0.769	0.638	

### Structural model assessment

With the satisfactory measurement model, the structural model was assessed to test the research hypotheses and estimate the relationships among the constructs.

#### Path coefficients and coefficient of determination (*R*^2^)

**Figure 2:**
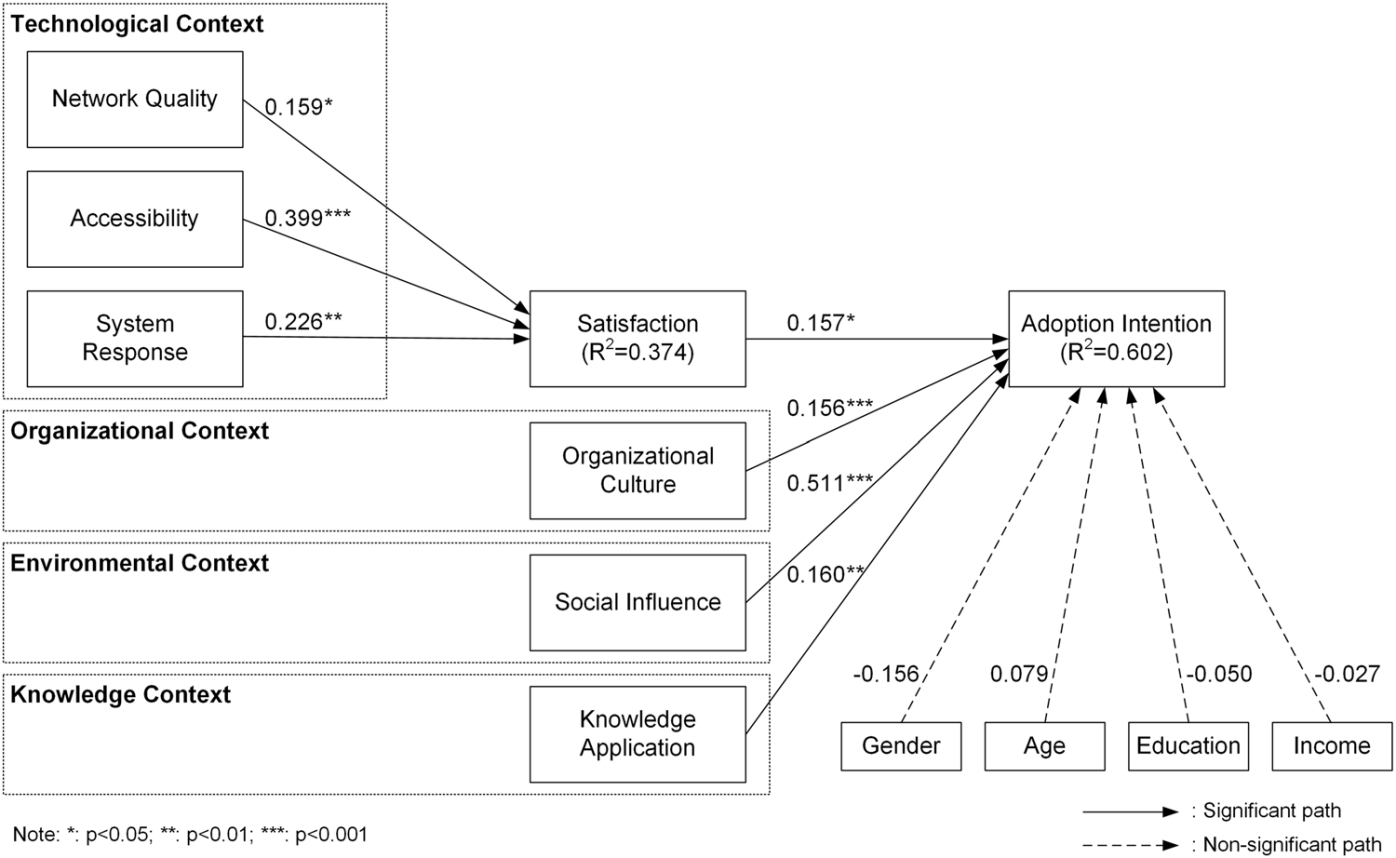
The path coefficients of the research model.

The path coefficients (*β*) and their corresponding t-values were obtained using the bootstrapping procedure with 5,000 resamples. In Fig. 2, the results show that network quality, accessibility, and system response are positively correlated with satisfaction. Furthermore, organizational culture, social influence, and knowledge application have a significant positive correlation with the intention of adopting ChatGPT among university students, which supports research hypotheses.

The outcomes for structural model are detailed in Table 6. In the results, a significant correlation was observed between network factors and satisfaction (*β﻿* = 0.159, *t* = 2.219), which aligns with H1. Consistent with H2, accessibility and satisfaction are significantly correlated (*β﻿* = 0.399, *t* = 6.625). A strong correlation was found between system response and satisfaction (*β﻿* = 0.226, *t* = 3.106), as stated in H3. As hypothesized in H4, there is a significant correlation between satisfaction and adoption intention (﻿*β﻿* = 0.157, *t* = 2.367). Organizational culture is positively correlated with adoption intention (﻿*β﻿* = 0.156, *t* = 3.968), supporting H5. Similarly, a notable correlation was found between social influence and adoption intention (﻿*β﻿* = 0.511, *t* = 9.831), which confirms H6. The correlation of knowledge application with adoption intention (*β﻿* = 0.160, *t* = 2.679) was also statistically significant, supporting H7. Regarding control variables, gender, age, education, and income do not have a correlation with adoption intention.

**Table 6:** Test results.

H	Variable 1	Variable 2	Coefficient﻿	*T* value	*P *value	Result
H1	Network quality	Satisfaction	0.159	2.219	0.027	Supported
H2	Accessibility	Satisfaction	0.399	6.625	0.000	Supported
H3	System response	Satisfaction	0.226	3.106	0.002	Supported
H4	Satisfaction	Adoption intention	0.157	2.367	0.018	Supported
H5	Organizational culture	Adoption Intention	0.156	3.968	0.000	Supported
H6	Social influence	Adoption intention	0.511	9.831	0.000	Supported
H7	Knowledge application	Adoption intention	0.160	2.679	0.007	Supported
CV	Gender	Adoption intention	− 0.156	1.906	0.057	Not Significant
CV	Age	Adoption intention	0.079	0.898	0.369	Not Significant
CV	Education	Adoption intention	− 0.050	0.913	0.361	Not Significant
CV	Income	Adoption intention	− 0.027	0.484	0.628	Not Significant

CV stands for control variable.

The *R*^2^ value for the adoption intention construct was calculated, which represents the proportion of variance explained by the predictor variables. The *R*^2^ value was 0.602, indicating that the model explained 60.2% of the variance in adoption intention, which is considered a moderate effect size^96^.

#### Effect size (*f*^2^)

The *f*^2^ values were computed to assess the effect size of each predictor on the adoption intention. All *f*^2^ values were above the threshold of 0.02, indicating small-to-medium effect sizes for the predictors^96^. Table 7 describes the *f*^2^ results.

**Table 7:** f^2^ results.

Constructs	1	2	3	4	5	6	7	8
1. Network				0.025				
2. Accessibility				0.224				
3. System response				0.050				
4. Satisfaction								0.035
5. Organizational culture								0.054
6. Social influence								0.444
7. Knowledge application								0.035
8. Adoption intention								

### Endogeneity

Endogeneity in SEM can introduce biases and can lead to incorrect interpretations of the relationships between variables. This challenge can emerge from factors like missing variables, inaccuracies in measurements, or when there is a two-way causal relationship between the predictor and outcome variables^97^. To counter potential endogeneity issues in our research, we utilized the Gaussian Copula Method within PLS-SEM via SmartPLS 4^98,99^. This technique helps in discerning a nonlinear interdependence between dependent and independent variables, thereby offering an in-depth comprehension of their associations. As shown in Table A2, no Gaussian copulas showed statistical significance in any of the model setups (*p* value > 0.05). All variables in our framework were classified as exogenous, meaning that there was no link to the error term within the set equations. This finding underscores that our data and model structure are not affected by endogeneity, lending further credence to the robustness of our structural model^98^.

## Discussion

The primary goal of this study was to explore the factors influencing adoption intention by integrating the knowledge aspect into the TOE framework.

Our findings suggest a significant correlation between network quality and satisfaction. This is in concordance with previous studies that have underscored the role of network quality in influencing user satisfaction across various technologies^60–62^. It stands to reason that an enhanced network quality can provide a seamless user experience, potentially leading to increased satisfaction. This aligns with the observation that students using ChatGPT benefit from a stable and high-quality network connection.

Additionally, the observed correlation between accessibility and satisfaction corroborates previous findings that emphasized the role of accessibility in increasing user satisfaction in technological contexts^43,65,100^. These studies collectively highlighted the indispensable role of accessibility in enhancing user contentment with technological platforms. In addition, a seamless user experience is often hinged on interfaces that are both intuitive and user-centric. Such interfaces facilitate smooth navigation, rapid information retrieval, and hassle-free downloads. It is evident that when users encounter minimal barriers in accessing desired content, their overall satisfaction with the platform is significantly boosted. In essence, optimizing accessibility is a cornerstone in ensuring a gratifying user journey in technological environments.

Expanding on our findings, it becomes evident that the efficacy of a system, particularly AI-driven ones like ChatGPT, is closely intertwined with its responsiveness. Our results distinctly mirror insights from earlier investigations, notably Uzir et al.^69^, wherein the agility and accuracy of AI system responses were central to user satisfaction. Delving deeper into the nuances of this correlation, we discern that users, especially in academic settings like universities, prioritize speed and precision. When these students interact with AI tools, they often seek instant, correct, and relevant feedback. Any delays or inaccuracies can disrupt their academic flow, potentially resulting in reduced reliance on such systems. Hence, for AI platforms catering to the academic segment, it is not just about having a response; it is about having the right response at the right time. This underscores the imperative for developers and educators to refine system agility and accuracy, ensuring that AI tools continue to be invaluable assets in educational settings.

Consistent with previous empirical findings, our study underscores the correlation between satisfaction and the intention to adopt AI-centric technologies like ChatGPT^73–75^. This nexus between satisfaction and adoption intention, especially among university students, is not merely transactional. It delves deeper into how AI interfaces resonate with their academic aspirations, usability expectations, and overall user experience. Given the rapidly evolving technological landscape, students are not just looking for functionality, but for seamless integration of utility and user-centric design. As these young adults navigate their academic endeavors, the satisfaction derived from using tools such as ChatGPT becomes paramount. This implies that for wider adoption, a holistic focus on cultivating and enhancing user satisfaction is indispensable.

In the context of our findings, the relationship between organizational culture and adoption intention becomes increasingly salient. Rooted in the intricate dynamics of how academic settings function, organizational culture emerges as a key influencer in shaping students' perceptions and intentions toward technological adoption. As highlighted by Heinze and Heinze^76^, when an academic institution nurtures an environment that fosters innovation, openness, and technological embrace, it implicitly encourages students to experiment with and adopt newer tools. This creates an underlying ecosystem where technologies like ChatGPT aren't just seen as external tools but become embedded into the academic fabric. Thus, an embracing organizational culture doesn't just support but actively amplifies the reach and acceptance of such AI platforms among its student body.

The realm of social influence, as our study elucidates, extends far beyond mere conformity. Within the digital landscape of today's universities, the choices of peers and influencers greatly mold individual decisions, especially in technology adoption. When peers commend or advocate for a certain technology, it carries a weight of validation that often transcends technical specifications or brand messaging. In line with findings from previous studies^80,81^, it is evident that social cues, shared experiences, and word-of-mouth play instrumental roles in shaping an individual's tech preferences. In the case of ChatGPT, positive feedback, discussions, or even casual mentions in academic settings can significantly drive its adoption rate. Essentially, the role of social interplay cannot be understated in determining the success or failure of technologies in academic environments.

In the contemporary academic landscape, where digitization and technological enhancements are paramount, the role of tools that facilitate knowledge application becomes more pronounced. Our study, delving deep into the correlation metrics, indicates a noteworthy bond between knowledge application and the intention behind adopting ChatGPT. Drawing parallels with Al-Sharafi et al.^57^, it is evident that when AI chatbots, like ChatGPT, are perceived not merely as tools for communication but also as potent mediums for knowledge assimilation and application, their acceptance becomes more widespread. Students, always on the lookout for efficient ways to augment their learning, recognize the multifaceted utility of ChatGPT. Whether it is for quick information retrieval, clarifying academic doubts, or even simulating intricate discussions, ChatGPT stands as a beacon of potential. It is this potent blend of AI efficiency with academic value that makes ChatGPT an attractive proposition for students who want to amplify their learning outcomes.

## Conclusion

### Theoretical contributions

This research provides insights into the factors influencing ChatGPT adoption among university students, encompassing technological, organizational, environmental, and knowledge dimensions. Notably, by integrating a knowledge context into the TOE framework, the study aims to enrich the current understanding and bridge potential gaps. While it offers a comprehensive view on AI language model adoption, it is essential to interpret these findings with a nuance that distinguishes correlation from causation.

While the study highlights the relevance of factors like network quality, accessibility, and system response in the context of ChatGPT adoption intentions, it is imperative to understand that the identified relationships are correlational. Therefore, direct causative actions based on these correlations should be undertaken with caution. Our findings echo previous research emphasizing the importance of various qualities—system, information, and service—in the adoption of AI tools^21,44,101^. Furthermore, the observed association between user satisfaction and adoption intention aligns with the literature on mobile learning technologies^26,28^, again highlighting the correlational nature of these relationships.

Our findings delve deeper into the intricate relationship between organizational culture and technology adoption, notably ChatGPT. Drawing parallels with the current literature concerning technology adoption in educational institutions^102–104^, we find a consistent narrative that underscores the role of a nurturing academic environment. There appears to be a synergistic interplay at work. In environments where the institutional culture is already oriented towards technology embracement, AI tools like ChatGPT stand a better chance of widespread acceptance. This underscores the importance of fostering a proactive tech-centric culture within educational settings, not just for the introduction but also for the seamless integration of novel technologies like ChatGPT.

Building upon the existing body of knowledge, our study meticulously underscores the significance of social influence in the adoption of ChatGPT, reflecting patterns observed in various other contexts as denoted by studies^23,105,106^. By zeroing in on the unique microcosm of the university setting, we have been able to add layers of depth and specificity to this broader narrative. Yet, in interpreting these findings, it's essential to approach them with a discerning lens. Rather than viewing them as definitive causative links, it would be more apt to perceive these patterns as markers pointing towards potential trends. This nuanced understanding paves the way for further exploration and corroborative research in diverse settings and demographics.

The alignment of students' perception of ChatGPT as a crucial tool for knowledge application with their intensified adoption tendencies sheds light on the broader tenets of technology adoption theories, particularly those focusing on perceived usefulness, as referenced in studies^25,107^. This relationship suggests that the more students discern the value in a technology, the more inclined they are to integrate it into their academic routines. However, while this observation holds merit, it's imperative that we don't rest on these laurels. Future research endeavors should sharpen their focus on various forms of knowledge application, discerning which specific types wield the most influence on the intention to adopt. Such granularity can pave the way for more tailored interventions and strategies to boost the adoption of tools like ChatGPT in educational settings.

The absence of notable effects of demographic factors such as age, gender, education, and income on the intent to adopt ChatGPT is a nuanced observation. While this lack of significance contradicts certain studies, as seen in reference^108^, it harmoniously aligns with others, like those in references^109,110^. A potential interpretation of these findings could be attributed to the universality of ChatGPT's design and its myriad features. The platform seems to be crafted in such a way that it transcends typical demographic boundaries, thereby resonating with a broad audience. This suggests that the inherent appeal and usability of ChatGPT are not restricted to specific groups, but rather it offers a user experience that can be appreciated and harnessed by diverse segments of the population.

### Practical implications

The findings of this study offer multiple insights into factors correlated with the intention to adopt ChatGPT among university students. These insights can guide universities, educational institutions, technology developers, and policymakers when considering strategies that might encourage the use of AI tools like ChatGPT to improve learning.

The observed correlation between network quality and satisfaction implies that a reliable network might influence student satisfaction. Universities could consider improving their network infrastructure, potentially enhancing the student experience while using ChatGPT. Collaborating with internet service providers for better connectivity and wider campus coverage might be a strategy worth exploring^111,112^. Similarly, the association between accessibility and satisfaction indicates potential benefits from making ChatGPT more accessible, either by embedding it into existing LMS or creating dedicated platforms^111,112^. Training sessions can help students maximize the potential of ChatGPT. The correlation between system response and satisfaction underscores the potential importance of the user experience. Although this does not establish causation, developers could contemplate refining ChatGPT's responsiveness, considering this association^113^. The relationship between satisfaction and adoption intention suggests that a positive user experience might be influential. Regular feedback from students and collaborations with developers might ensure a more gratifying interaction with ChatGPT^113^.

The intricate relationship between organizational culture and adoption intention provides valuable insights for institutions aiming to incorporate advanced technological tools. Delving deeper into this association, it is evident that establishing a tech-friendly university culture is not merely about introducing students to AI tools but is about instilling a sense of technological appreciation throughout the institution. While tools like ChatGPT are at the forefront of AI-driven educational advancements, a successful implementation relies heavily on the organizational ethos^114,115^. Hence, universities should aim for an all-encompassing approach. This might include integrating AI tools into diverse courses—from liberal arts to STEM subjects—ensuring faculty is not only trained but also actively engaged in AI tool pedagogy, and perhaps most significantly, organizing events that not just showcase ChatGPT's potential, but also spark intellectually stimulating discussions on the future of AI in education.

Furthermore, the significant influence of social factors on adoption intention cannot be underestimated. It is human nature to trust and lean on peers for recommendations, especially when navigating unfamiliar terrains like advanced technology. Universities can harness this by creating environments that facilitate organic dialogues about AI tools. Platforms that encourage students to share their experiences, testimonials, or even challenges with ChatGPT can be pivotal. Additionally, formalizing this through ambassador programs where selected students guide and mentor their peers on the nuances of using ChatGPT can be a game-changer, especially when we remember that these insights are primarily correlative.

Lastly, while theoretical knowledge about AI tools like ChatGPT is essential, practical, real-world application truly anchors learning. The observed correlation between knowledge application and adoption intention is a clear call for educators to get creative. Institutions could curate events such as case study competitions centered on ChatGPT or perhaps even introduce semester-long projects that require students to use ChatGPT in solving real-world problems. By allowing students to see first-hand the utility and relevance of ChatGPT, universities can significantly influence the rate and enthusiasm of its adoption among their students.

In all these considerations, it is crucial for stakeholders to remember that the study identifies relationships and not direct causation. Thus, any strategic actions based on these insights should be taken judiciously and be open to iterative refinement.

### Limitation and further research

This study encompasses several limitations that warrant acknowledgment, and these very limitations also pave the way for intriguing future research opportunities. First, a significant limitation of our study lies in its lack of attention to the social considerations associated with the adoption of AI-driven tools in education. While we concentrated on the technological, organizational, and environmental factors influencing adoption, it is imperative to acknowledge the broader societal implications. Issues such as data privacy, the potential for algorithmic bias, and the chance that AI tools might exacerbate educational inequalities are critical elements that deserve attention. Future research should expand to include these considerations, providing a comprehensive perspective on the challenges and opportunities of AI adoption in education. Second, the present study refrained from investigating potential moderating influences such as individual traits, prior experience with AI-centric tools, or any preliminary interactions with ChatGPT. There lies a research prospect in understanding how such factors might impact the correlation between pivotal determinants and the intention to adopt. In addition, we acknowledge that the study’s design was correlational, limiting our ability to make causal inferences. To address this critical limitation, we propose the conduct of experimental or longitudinal studies in future endeavors. This approach can help in manipulating specific variables (for instance, network quality) and observing their direct implications on adoption intention across a timespan. Third, we overlooked the incorporation of ethical considerations, even though such aspects can play a pivotal role in the adoption intention. Future studies should emphasize and integrate ethical dimensions, especially in the context of AI-driven tools, to provide a more comprehensive understanding of user adoption patterns. Lastly, a clear limitation of this study is its dependence on subjective measures without the inclusion of objective performance data. Future research should contemplate supplementing self-reported data with objective metrics, such as network quality statistics and actual usage duration, to offer a more comprehensive and unbiased perspective on the determinants influencing adoption intention.

## Summary

Our study embarked on a mission to understand the adoption intention of ChatGPT among university students, emphasizing a variety of variables such as network quality, system response, satisfaction, organizational culture, social influence, knowledge application, and demographic factors. By delving into the complexities of student behavior, we identified that system functionality, network stability, and accessibility are of utmost importance to students when evaluating ChatGPT as a trustworthy tool. Furthermore, satisfaction and organizational culture—reflecting the university environment—the influence of social peers, and the practical application of knowledge from using ChatGPT significantly influenced adoption intention.

Several demographic variables, including age, gender, and education level, nuanced the adoption narrative, each exerting varying degrees of influence. These findings not only serve as empirical indicators but also act as essential guideposts for developers, educators, and institutions to comprehend, adjust to, and advocate for tools like ChatGPT more effectively.

As with any research, our study had limitations. Nonetheless, it lays the groundwork for future research to delve deeper into the cultural dimensions and other inherent factors impacting adoption intention. The culmination of our findings highlights the importance of system robustness, satisfaction with ChatGPT, an organizational culture that promotes the use of ChatGPT by students, the importance of peer influence in adoption, and the vital need to guarantee uninterrupted access and reliability for users.

### Supplementary Information

Supplementary Tables.

## Data Availability

The datasets used and/or analyzed during the current study available from the corresponding author on reasonable request.

## References

[1] Chassignol M, Khoroshavin A, Klimova A, Bilyatdinova A (2018). Artificial Intelligence trends in education: A narrative overview. Proc. Comput. Sci..

[2] Rudolph J, Tan S, Tan S (2023). ChatGPT: Bullshit spewer or the end of traditional assessments in higher education?. J. Appl. Learn. Teach..

[3] Haleem A, Javaid M, Singh RP (2022). An era of ChatGPT as a significant futuristic support tool: A study on features, abilities, and challenges. BenchCouncil Trans. Benchmarks, Stand. Eval..

[4] Sullivan M, Kelly A, McLaughlan P (2023). ChatGPT in higher education: Considerations for academic integrity and student learning. J. Appl. Learn. Teach..

[5] Paul J, Ueno A, Dennis C (2023). ChatGPT and consumers: Benefits, pitfalls and future research agenda. Int. J. Consum. Stud..

[6] Castelvecchi D (2022). Are ChatGPT and AlphaCode going to replace programmers?. Nature.

[7] Adiguzel T, Kaya MH, Cansu FK (2023). Revolutionizing education with AI: Exploring the transformative potential of ChatGPT. Contemp. Educ. Technol..

[8] AlAfnan MA, Dishari S, Jovic M, Lomidze K (2023). Chatgpt as an educational tool: Opportunities, challenges, and recommendations for communication, business writing, and composition courses. J. Artif. Intell. Technol..

[9] Jo H (2023). Decoding the ChatGPT mystery: A comprehensive exploration of factors driving AI language model adoption. Inf. Dev..

[10] Ansong E, Lovia Boateng S, Boateng R (2017). Determinants of E-learning adoption in universities: Evidence from a developing country. J. Educ. Technol. Syst..

[11] Salimon MG (2021). Malaysian SMEs m-commerce adoption: TAM 3, UTAUT 2 and TOE approach. J. Sci. Technol. Policy Manag..

[12] Tornatzky LG, Fleischer M, Chakrabarti AK (1990). Processes of Technological Innovation.

[13] Firat M (2023). How Chat GPT can Transform Autodidactic Experiences and Open Education.

[14] Rogers EM (1995). Diffusion of innovation.

[15] Biswas, S. 223312 (Radiological Society of North America, 2023).

[16] Surameery NMS, Shakor MY (2023). Use chat gpt to solve programming bugs. Int. J. Inf. Technol. Comput. Eng. (IJITC).

[17] Shen, M. *How to Fix ChatGPT Network Error: Solutions and Tips*, <https://www.awesomescreenshot.com/blog/knowledge/chatgpt-network-error> (2023).

[18] DeLone WH, McLean ER (2003). The DeLone and McLean model of information systems success: A ten-year update. J. Manag. Inf. Syst..

[19] Abidi, Y. *ChatGPT History Gone: How to Retrieve Your Lost ChatGPT History*, <https://www.makeuseof.com/how-to-retreive-chatgpt-history/> (2023).

[20] Sidhu, K. *OpenAI ChatGPT users Report Login Loop or Internal Server Error (Potential Workaround)*, <https://piunikaweb.com/2023/04/25/open-ai-chatgpt-users-report-login-loop-or-internal-server-error/> (2023).

[21] Nicolescu L, Tudorache MT (2022). Human-computer interaction in customer service: The experience with AI chatbots—a systematic literature review. Electronics.

[22] Schein EH (2010). Organizational culture and leadership.

[23] Kuberkar S, Singhal TK (2020). Factors influencing adoption intention of AI powered chatbot for public transport services within a smart city. Int. J. Emerg. Technol. Learn..

[24] Terblanche N, Cilliers D (2020). Factors that influence users’ adoption of being coached by an artificial intelligence coach. Philos. Coach.: Int. J..

[25] Davis FD (1989). Perceived usefulness, perceived ease of use, and user acceptance of information technology. MIS Q..

[26] Ashfaq M, Yun J, Yu S, Loureiro SMC (2020). I, Chatbot: Modeling the determinants of users’ satisfaction and continuance intention of AI-powered service agents. Telemat. Inform..

[27] Eren BA (2021). Determinants of customer satisfaction in chatbot use: Evidence from a banking application in Turkey. Int. J. Bank Mark..

[28] Nguyen DM, Chiu Y-TH, Le HD (2021). Determinants of continuance intention towards banks’ chatbot services in Vietnam: A necessity for sustainable development. Sustainability.

[29] Biswas S (2023). Prospective role of Chat GPT in the military: According to ChatGPT. Qeios.

[30] Kung TH (2023). Performance of ChatGPT on USMLE: Potential for AI-assisted medical education using large language models. PLOS Digital Health.

[31] George AS, George AH (2023). A review of ChatGPT AI's impact on several business sectors. Partn. Univ. Int. Innov. J..

[32] Farrokhnia M, Banihashem SK, Noroozi O, Wals A (2023). A SWOT analysis of ChatGPT: Implications for educational practice and research. Innov. Educ. Teach. Int..

[33] Cotton DRE, Cotton PA, Shipway JR (2023). Chatting and cheating: Ensuring academic integrity in the era of ChatGPT. Innov. Educ. Teach. Int..

[34] Zhai, X. *ChatGPT: Artificial Intelligence for Education*. (2022).

[35] Nguyen QN, Ta A, Prybutok V (2019). An integrated model of voice-user interface continuance intention: The gender effect. Int. J. Hum.-Comput. Interact..

[36] Balakrishnan J, Abed SS, Jones P (2022). The role of meta-UTAUT factors, perceived anthropomorphism, perceived intelligence, and social self-efficacy in chatbot-based services?. Technol. Forecast. Soc. Change.

[37] Ikumoro AO, Jawad MS (2019). Intention to use intelligent conversational agents in e-commerce among Malaysian SMEs: An integrated conceptual framework based on tri-theories including unified theory of acceptance, use of technology (UTAUT), and TOE. Int. J. Acad. Res. Bus. Soc. Sci..

[38] Rodríguez Cardona, D., Werth, O., Schönborn, S. & Breitner, M. H. A mixed methods analysis of the adoption and diffusion of Chatbot Technology in the German insurance sector. (2019).

[39] Mokhtar, S. S. M. & Salimon, M. G. in *Marketing Communications and Brand Development in Emerging Markets Volume II: Insights for a Changing World* 25–53 (Springer, 2022).

[40] Ojiaku OC, Osarenkhoe A (2018). Determinants of customers’ brand choice and continuance intentions with mobile data service provider: The role of past experience. Glob. Bus. Rev..

[41] Ting H (2020). What determines customers' loyalty towards telecommunication service? Mediating roles of satisfaction and trust. Int. J. Serv., Econ. Manag..

[42] Almaiah MA, Man M (2016). Empirical investigation to explore factors that achieve high quality of mobile learning system based on students’ perspectives. Eng. Sci. Technol., Int. J..

[43] Pituch KA, Lee Y-K (2006). The influence of system characteristics on e-learning use. Comput. Educ..

[44] Jiang, J. & Ahuja, N. in *Proceedings of the 43rd International ACM SIGIR Conference on Research and Development in Information Retrieval.*

[45] Gupta A, Yousaf A, Mishra A (2020). How pre-adoption expectancies shape post-adoption continuance intentions: An extended expectation-confirmation model. Int. J. Inf. Manag..

[46] Pan S, Jordan-Marsh M (2010). Internet use intention and adoption among Chinese older adults: From the expanded technology acceptance model perspective. Comput. Hum. Behav..

[47] Al-Emadi, K. A., Kassim, Z. A. & Razzaque, A. in *Innovative Strategies for Implementing FinTech in Banking* 291–301 (IGI Global, 2021).

[48] Chen J-S, Le T-T-Y, Florence D (2021). Usability and responsiveness of artificial intelligence chatbot on online customer experience in E-retailing. Int. J. Retail Distrib. Manag..

[49] Abdulkarem A, Hou W (2021). The impact of organizational context on the levels of cross-border E-commerce adoption in Chinese SMEs: The moderating role of environmental context. J. Theor. Appl. Electron. Commer. Res..

[50] Baker, J. in *Information Systems Theory: Explaining and Predicting Our Digital Society, Vol. 1* (eds Yogesh K. Dwivedi, Michael R. Wade, & Scott L. Schneberger) 231–245 (Springer, 2012).

[51] Adamy P, Heinecke W (2005). The influence of organizational culture on technology integration in teacher education. J. Technol. Teach. Educ..

[52] Hong KH (2010). CALL teacher education as an impetus for L2 teachers in integrating technology. ReCALL.

[53] Romm T, Pliskin N, Weber Y, Lee AS (1991). Identifying organizational culture clash in MIS implementation: When is it worth the effort?. Inf. Manag..

[54] Deutsch M, Gerard HB (1955). A study of normative and informational social influences upon individual judgment. J. Abnorm. Soc. Psychol..

[55] Cao G, Duan Y, Edwards JS, Dwivedi YK (2021). Understanding managers’ attitudes and behavioral intentions towards using artificial intelligence for organizational decision-making. Technovation.

[56] Al-Emran M, AlQudah AA, Abbasi GA, Al-Sharafi MA, Iranmanesh M (2023). Determinants of using AI-based chatbots for knowledge sharing: Evidence from PLS-SEM and fuzzy sets (fsQCA). IEEE Trans. Eng. Manag..

[57] Al-Sharafi MA (2022). Understanding the impact of knowledge management factors on the sustainable use of AI-based chatbots for educational purposes using a hybrid SEM-ANN approach. Interact. Learn. Environ..

[58] Azouzi, R. E., Altman, E. & Wynter, L. in *Teletraffic Science and Engineering* Vol. 5 (eds J. Charzinski, R. Lehnert, & P. Tran-Gia) 369–378 (Elsevier, 2003).

[59] Shafei I, Tabaa H (2016). Factors affecting customer loyalty for mobile telecommunication industry. EuroMed. J. Bus..

[60] Pavlou PA, Fygenson M (2006). Understanding and predicting electronic commerce adoption: An extension of the theory of planned behavior. MIS Q..

[61] Finley B (2017). Does network quality matter? A field study of mobile user satisfaction. Pervasive Mobile Comput..

[62] Ting H (2020). What determines customers' loyalty towards telecommunication service Mediating roles of satisfaction and trust. Int. J. Serv., Econ. Manag..

[63] Zhou R (2019). Measuring e-service quality and its importance to customer satisfaction and loyalty: An empirical study in a telecom setting. Electron. Commer. Res..

[64] Ahn T, Ryu S, Han I (2007). The impact of web quality and playfulness on user acceptance of online retailing. Inf. Manag..

[65] Shin D-H (2007). User acceptance of mobile internet: Implication for convergence technologies. Interact. Comput..

[66] Chen P-Y, Hitt LM (2002). Measuring switching costs and the determinants of customer retention in internet-enabled businesses: A study of the online brokerage industry. Inf. Syst. Res..

[67] Lin H-H, Wang Y-S (2006). An examination of the determinants of customer loyalty in mobile commerce contexts. Inf. Manag..

[68] Ramadiani Azainil, Haryaka U, Agus F, Kridalaksana AH (2017). User satisfaction model for E-learning using smartphone. Proc. Comput. Sci..

[69] Uzir MUH (2021). Applied Artificial Intelligence and user satisfaction: Smartwatch usage for healthcare in Bangladesh during COVID-19. Technol. Soc..

[70] Roca JC, Chiu C-M, Martínez FJ (2006). Understanding E-learning continuance intention: An extension of the technology acceptance model. Int. J. Hum.-Comput. Stud..

[71] Vărzaru AA, Bocean CG, Rotea CC, Budică-Iacob A-F (2021). Assessing antecedents of behavioral intention to use mobile technologies in E-commerce. Electronics.

[72] Dai J, Li R, Liu Z (2021). Does initial experience affect consumers’ intention to use autonomous vehicles? Evidence from a field experiment in Beijing. Accid. Anal. Prev..

[73] Cheng Y, Jiang H (2020). How do AI-driven chatbots impact user experience? Examining gratifications, perceived privacy risk, satisfaction, loyalty, and continued use. J. Broadcast. Electron. Media.

[74] Pal D, Babakerkhell MD, Zhang X (2021). Exploring the determinants of users’ continuance usage intention of smart voice assistants. IEEE Access.

[75] Han S, Yang H (2018). Understanding adoption of intelligent personal assistants: A parasocial relationship perspective. Ind. Manag. Data Syst..

[76] Heinze KL, Heinze JE (2020). Individual innovation adoption and the role of organizational culture. Rev. Manag. Sci..

[77] Roos, J. *ChatGPT: The Next Firestorm in Education* <https://www.aacsb.edu/insights/articles/2023/02/chatgpt-the-next-firestorm-in-education> (2023).

[78] Yuen AHK, Ma WWK (2008). Exploring teacher acceptance of e-learning technology. Asia-Pac. J. Teach. Educ..

[79] Venkatesh V, Morris MG, Davis GB, Davis FD (2003). User acceptance of information technology: Toward a unified view. MIS Q..

[80] Sun P-C, Tsai RJ, Finger G, Chen Y-Y, Yeh D (2008). What drives a successful E-learning? An empirical investigation of the critical factors influencing learner satisfaction. Comput. Educ..

[81] Alalwan AA, Dwivedi YK, Rana NP (2017). Factors influencing adoption of mobile banking by Jordanian bank customers: Extending UTAUT2 with trust. Int. J. Inf. Manag..

[82] Fang YH, Chiu CM, Wang ETG (2011). Understanding customers' satisfaction and repurchase intentions. Internet Res..

[83] Yi-Hsuan L, Yi-Chuan H, Chia-Ning H (2011). Adding innovation diffusion theory to the technology acceptance model: Supporting employees' intentions to use E-learning systems. J. Educ. Technol. Soc..

[84] Dwivedi YK (2023). “So what if ChatGPT wrote it?” Multidisciplinary perspectives on opportunities, challenges and implications of generative conversational AI for research, practice and policy. Int. J. Inf. Manag..

[85] Alavi M, Leidner DE (2001). Knowledge management and knowledge management systems: Conceptual foundations and research issues. MIS Q..

[86] Wang W-T, Wang C-C (2009). An empirical study of instructor adoption of web-based learning systems. Comput. Educ..

[87] Arnott D, Pervan G (2014). A critical analysis of decision support systems research revisited: The rise of design science. J. Inf. Technol..

[88] Al-Emran M, Elsherif HM, Shaalan K (2016). Investigating attitudes towards the use of mobile learning in higher education. Comput. Hum. Behav..

[89] Lund BD (2023). ChatGPT and a new academic reality: Artificial Intelligence-written research papers and the ethics of the large language models in scholarly publishing. J. Assoc. Inf. Sci. Technol..

[90] Soper, D. *Free Statistics Calculators* <https://www.danielsoper.com/statcalc/default.aspx> (2023).

[91] Hair J, Hollingsworth CL, Randolph AB, Chong AYL (2017). An updated and expanded assessment of PLS-SEM in information systems research. Ind. Manag. Data Syst..

[92] Podsakoff PM, MacKenzie M, Scott B, Lee J-Y, Podsakoff NP (2003). Common method biases in behavioral research: A critical review of the literature and recommended remedies. J. Appl. Psychol..

[93] Lindell MK, Whitney DJ (2001). Accounting for common method variance in cross-sectional research designs. J. Appl. Psychol..

[94] Kock N (2015). Common method bias in PLS-SEM: A full collinearity assessment approach. Int. J. E-Collab. (IJEC).

[95] Fornell C, Larcker DF (1981). Evaluating structural equation models with unobservable variables and measurement error. J. Mark. Res..

[96] Cohen J (1992). A power primer. Psychol. Bull..

[97] Antonakis J, Bendahan S, Jacquart P, Lalive R (2010). On making causal claims: A review and recommendations. Leadersh. Q..

[98] Hult GTM (2018). Addressing endogeneity in international marketing applications of partial least squares structural equation modeling. J. Int. Mark..

[99] Joe H (2014). Dependence Modeling with Copulas.

[100] Islam AN (2014). Sources of satisfaction and dissatisfaction with a learning management system in post-adoption stage: A critical incident technique approach. Comput. Hum. Behav..

[101] Adamopoulou, E. & Moussiades, L. 373–383 (Springer International Publishing).

[102] Maragno G, Tangi L, Gastaldi L, Benedetti M (2022). AI as an organizational agent to nurture: Effectively introducing chatbots in public entities. Public Manag. Rev..

[103] Rahim NIM, Iahad NA, Yusof AF, Al-Sharafi MA (2022). AI-based chatbots adoption model for higher-education institutions: A hybrid PLS-SEM-neural network modelling approach. Sustainability.

[104] Zhang JJY, Følstad A, Bjørkli CA (2023). Organizational factors affecting successful implementation of chatbots for customer service. J. Internet Commer..

[105] Cheng M, Li X, Xu J (2022). Promoting healthcare worker’s adoption intention of artificial-intelligence-assisted diagnosis and treatment: The chain mediation of social influence and human-computer trust. Int. J. Environ. Res. Public Health.

[106] Huang CY, Yang MC, Huang CY (2021). An empirical study on factors influencing consumer adoption intention of an AI-powered chatbot for health and weight management. Int. J. Perform. Eng..

[107] Scherer R, Siddiq F, Tondeur J (2019). The technology acceptance model (TAM): A meta-analytic structural equation modeling approach to explaining teachers’ adoption of digital technology in education. Comput. Educ..

[108] Venkatesh V, Thong JYL, Xu X (2012). Consumer acceptance and use of information technology: Extending the unified theory of acceptance and use of technology. MIS Q..

[109] Al-Emran M, Mezhuyev V, Kamaludin A (2018). Technology acceptance model in M-learning context: A systematic review. Comput. Educ..

[110] Dwivedi YK (2021). Artificial Intelligence (AI): Multidisciplinary perspectives on emerging challenges, opportunities, and agenda for research, practice and policy. Int. J. Inf. Manag..

[111] Al-Busaidi KA, Al-Shihi H (2010). Instructors' acceptance of learning management systems: A theoretical framework. Commun. IbIMA.

[112] Ilyas M, Kadir KA, Adnan Z (2017). Demystifying the learning management system (LMS): Journey from E-learning to the strategic role. Eur. J. Bus. Manag..

[113] Shafer, L. *Making Student Feedback Work*, <https://www.gse.harvard.edu/news/uk/17/11/making-student-feedback-work> (2017).

[114] George DR, Dellasega C (2011). Use of social media in graduate-level medical humanities education: Two pilot studies from Penn state college of medicine. Med. Teach..

[115] Parry, G. *Artificial intelligence (AI) in Education*, <https://www.gsineducation.com/blog/artificial-intelligence-ai-in-education> (2023).

