# Supplementary material for: Analyzing ChatGPT adoption drivers with the TOEK framework
Source: Sci Rep. 2023 Dec 19;13:22606. doi: 10.1038/s41598-023-49710-0 (PMC10730566; doi:10.1038/s41598-023-49710-0)
Supplement: Supplementary file 1 — Supplementary Tables. [file 41598_2023_49710_MOESM1_ESM.docx]

# Appendix

Table A1. List of Constructs and Items

| Construct | Items | Mean | Source |
| --- | --- | --- | --- |
| Network  Quality | NEQ1 | The network speed of ChatGPT is good. | [Ojiaku and Osarenkhoe (2018)](#_ENREF_67) |
|  | NEQ2 | The network connection of ChatGPT is always available. |  |
|  | NEQ3 | The network connection of ChatGPT is reliable. |  |
| Accessibility | ACS1 | The provided contents on ChatGPT are easily accessible. | [Almaiah and Man (2016)](#_ENREF_14) |
|  | ACS2 | The provided contents on ChatGPT are easily downloadable. |  |
|  | ACS3 | The provided contents on ChatGPT are easily retrievable. |  |
| System  Response | SRP1 | When I am using ChatGPT, the system response is fast. | [Pituch and Lee (2006)](#_ENREF_74) |
|  | SRP2 | In general, the response time of ChatGPT is consistent. |  |
|  | SRP3 | In general, the response time of ChatGPT is reasonable. |  |
| Satisfaction | SAT1 | I am very satisfied with ChatGPT. | [Kim et al. (2016 telematics)](#_ENREF_54) |
|  | SAT2 | ChatGPT meets my expectations. |  |
|  | SAT3 | ChatGPT fits my needs/wants. |  |
| Organizational  Culture | OGC1 | Our university supports students using ChatGPT. | [Romm et al. (1991)](#_ENREF_80) |
|  | OGC2 | Our university promotes the use of ChatGPT through incentive programs. |  |
|  | OGC3 | Our university policy has a positive effect on the use of ChatGPT. |  |
| Social  Influence | SOI1 | People who influence me think that I should use ChatGPT. | [Venkatesh et al. (2012)](#_ENREF_101) |
|  | SOI2 | People who are important to me think I should use ChatGPT. |  |
|  | SOI3 | Most people who are important to me understand that I use ChatGPT. |  |
| Knowledge  Application | KAP1 | ChatGPT provides me with instant access to various types of knowledge. | [Al-Sharafi et al. (2022)](#_ENREF_11) |
|  | KAP2 | ChatGPT allows me to integrate different types of knowledge. |  |
|  | KAP3 | ChatGPT helps you better master the knowledge you learn in university. |  |
| Adoption  Intention | ADI1 | I intend to use ChatGPT. | [Davis (1989)](#_ENREF_31) |
|  | ADI2 | I expect that I would use ChatGPT in the future. |  |
|  | ADI3 | I plan to use ChatGPT in the future. |  |

Table A2. Assessment of endogeneity using the Gaussian Copula approach

| Test | Construct | Coefficient | *p* value |
| --- | --- | --- | --- |
| Gaussian copulas of model 1  (endogenous variable: SAT; outcome variable: NEQ) | NEQ | -0.079 | 0.813 |
|  | ACS | 0.393 | 0.000 |
|  | SRP | 0.222 | 0.002 |
|  | SAT | 0.157 | 0.018 |
|  | OGC | 0.156 | 0.000 |
|  | SOI | 0.511 | 0.000 |
|  | KAP | 0.160 | 0.007 |
|  | Gender | -0.156 | 0.057 |
|  | Age | 0.079 | 0.369 |
|  | Education | -0.050 | 0.361 |
|  | Income | -0.027 | 0.628 |
|  | ^GC^NEQ | 0.230 | 0.446 |
| Gaussian copulas of model 2  (endogenous variable: SAT; outcome variable: ACS) | NEQ | 0.158 | 0.028 |
|  | ACS | 0.264 | 0.348 |
|  | SRP | 0.225 | 0.002 |
|  | SAT | 0.157 | 0.018 |
|  | OGC | 0.156 | 0.000 |
|  | SOI | 0.511 | 0.000 |
|  | KAP | 0.160 | 0.007 |
|  | Gender | -0.156 | 0.057 |
|  | Age | 0.079 | 0.369 |
|  | Education | -0.050 | 0.361 |
|  | Income | -0.027 | 0.628 |
|  | ^GC^ACS | 0.112 | 0.615 |
| Gaussian copulas of model 3  (endogenous variable: SAT; outcome variable: SRP) | NEQ | 0.163 | 0.026 |
|  | ACS | 0.385 | 0.000 |
|  | SRP | -0.256 | 0.383 |
|  | SAT | 0.157 | 0.018 |
|  | OGC | 0.156 | 0.000 |
|  | SOI | 0.511 | 0.000 |
|  | KAP | 0.160 | 0.007 |
|  | Gender | -0.156 | 0.057 |
|  | Age | 0.079 | 0.369 |
|  | Education | -0.050 | 0.361 |
|  | Income | -0.027 | 0.628 |
|  | ^GC^SRP | 0.449 | 0.090 |
| Gaussian copulas of model 4  (endogenous variable: SAT; outcome variable: NEQ and ACS) | NEQ | -0.067 | 0.842 |
|  | ACS | 0.278 | 0.326 |
|  | SRP | 0.221 | 0.002 |
|  | SAT | 0.157 | 0.018 |
|  | OGC | 0.156 | 0.000 |
|  | SOI | 0.511 | 0.000 |
|  | KAP | 0.160 | 0.007 |
|  | Gender | -0.156 | 0.057 |
|  | Age | 0.079 | 0.369 |
|  | Education | -0.050 | 0.361 |
|  | Income | -0.027 | 0.628 |
|  | ^GC^NEQ | 0.218 | 0.477 |
|  | ^GC^ACS | 0.096 | 0.673 |
| Gaussian copulas of model 5  (endogenous variable: SAT; outcome variable: NEQ and SRP) | NEQ | 0.018 | 0.958 |
|  | ACS | 0.382 | 0.000 |
|  | SRP | -0.220 | 0.460 |
|  | SAT | 0.157 | 0.018 |
|  | OGC | 0.156 | 0.000 |
|  | SOI | 0.511 | 0.000 |
|  | KAP | 0.160 | 0.007 |
|  | Gender | -0.156 | 0.057 |
|  | Age | 0.079 | 0.369 |
|  | Education | -0.050 | 0.361 |
|  | Income | -0.027 | 0.628 |
|  | ^GC^NEQ | 0.140 | 0.643 |
|  | ^GC^SRP | 0.414 | 0.123 |
| Gaussian copulas of model 6  (endogenous variable: SAT; outcome variable: ACS and SRP) | NEQ | 0.163 | 0.027 |
|  | ACS | 0.351 | 0.224 |
|  | SRP | -0.245 | 0.430 |
|  | SAT | 0.157 | 0.018 |
|  | OGC | 0.156 | 0.000 |
|  | SOI | 0.511 | 0.000 |
|  | KAP | 0.160 | 0.007 |
|  | Gender | -0.156 | 0.057 |
|  | Age | 0.079 | 0.369 |
|  | Education | -0.050 | 0.361 |
|  | Income | -0.027 | 0.628 |
|  | ^GC^ACS | 0.029 | 0.903 |
|  | ^GC^SRP | 0.439 | 0.118 |
| Gaussian copulas of model 7  (endogenous variable: SAT; outcome variable: ADI) | NEQ | 0.159 | 0.027 |
|  | ACS | 0.399 | 0.000 |
|  | SRP | 0.226 | 0.002 |
|  | SAT | 0.253 | 0.342 |
|  | OGC | 0.155 | 0.000 |
|  | SOI | 0.514 | 0.000 |
|  | KAP | 0.157 | 0.012 |
|  | Gender | -0.157 | 0.057 |
|  | Age | 0.080 | 0.352 |
|  | Education | -0.054 | 0.328 |
|  | Income | -0.025 | 0.662 |
|  | ^GC^SAT | -0.086 | 0.686 |
| Gaussian copulas of model 8  (endogenous variable: ADI; outcome variable: OGC) | NEQ | 0.159 | 0.027 |
|  | ACS | 0.399 | 0.000 |
|  | SRP | 0.226 | 0.002 |
|  | SAT | 0.155 | 0.019 |
|  | OGC | -0.043 | 0.879 |
|  | SOI | 0.509 | 0.000 |
|  | KAP | 0.162 | 0.006 |
|  | Gender | -0.157 | 0.057 |
|  | Age | 0.075 | 0.400 |
|  | Education | -0.049 | 0.382 |
|  | Income | -0.028 | 0.624 |
|  | ^GC^OGC | 0.204 | 0.483 |
| Gaussian copulas of model 9  (endogenous variable: ADI; outcome variable: SOI) | NEQ | 0.159 | 0.027 |
|  | ACS | 0.399 | 0.000 |
|  | SRP | 0.226 | 0.002 |
|  | SAT | 0.159 | 0.017 |
|  | OGC | 0.157 | 0.000 |
|  | SOI | 0.571 | 0.000 |
|  | KAP | 0.158 | 0.009 |
|  | Gender | -0.157 | 0.057 |
|  | Age | 0.079 | 0.368 |
|  | Education | -0.052 | 0.344 |
|  | Income | -0.024 | 0.675 |
|  | ^GC^SOI | -0.052 | 0.671 |
| Gaussian copulas of model 10  (endogenous variable: ADI; outcome variable: KAP) | NEQ | 0.159 | 0.027 |
|  | ACS | 0.399 | 0.000 |
|  | SRP | 0.226 | 0.002 |
|  | SAT | 0.160 | 0.016 |
|  | OGC | 0.157 | 0.000 |
|  | SOI | 0.513 | 0.000 |
|  | KAP | 0.320 | 0.114 |
|  | Gender | -0.155 | 0.059 |
|  | Age | 0.077 | 0.378 |
|  | Education | -0.047 | 0.392 |
|  | Income | -0.028 | 0.612 |
|  | ^GC^KAP | -0.149 | 0.426 |
| Gaussian copulas of model 11  (endogenous variable: ADI; outcome variable: SAT and OGC) | NEQ | 0.159 | 0.027 |
|  | ACS | 0.399 | 0.000 |
|  | SRP | 0.226 | 0.002 |
|  | SAT | 0.263 | 0.329 |
|  | OGC | -0.058 | 0.845 |
|  | SOI | 0.512 | 0.000 |
|  | KAP | 0.158 | 0.010 |
|  | Gender | -0.158 | 0.057 |
|  | Age | 0.076 | 0.383 |
|  | Education | -0.052 | 0.344 |
|  | Income | -0.025 | 0.661 |
|  | ^GC^SAT | -0.097 | 0.653 |
|  | ^GC^OGC | 0.218 | 0.468 |
| Gaussian copulas of model 12  (endogenous variable: ADI; outcome variable: SAT and SOI) | NEQ | 0.159 | 0.027 |
|  | ACS | 0.399 | 0.000 |
|  | SRP | 0.226 | 0.002 |
|  | SAT | 0.237 | 0.399 |
|  | OGC | 0.156 | 0.000 |
|  | SOI | 0.558 | 0.000 |
|  | KAP | 0.156 | 0.013 |
|  | Gender | -0.157 | 0.058 |
|  | Age | 0.080 | 0.354 |
|  | Education | -0.054 | 0.323 |
|  | Income | -0.023 | 0.690 |
|  | ^GC^SAT | -0.070 | 0.754 |
|  | ^GC^SOI | -0.038 | 0.767 |
| Gaussian copulas of model 13  (endogenous variable: ADI; outcome variable: SAT and KAP) | NEQ | 0.159 | 0.027 |
|  | ACS | 0.399 | 0.000 |
|  | SRP | 0.226 | 0.002 |
|  | SAT | 0.196 | 0.494 |
|  | OGC | 0.157 | 0.000 |
|  | SOI | 0.513 | 0.000 |
|  | KAP | 0.305 | 0.178 |
|  | Gender | -0.156 | 0.060 |
|  | Age | 0.078 | 0.369 |
|  | Education | -0.049 | 0.380 |
|  | Income | -0.027 | 0.633 |
|  | ^GC^SAT | -0.032 | 0.888 |
|  | ^GC^KAP | -0.135 | 0.499 |
| Gaussian copulas of model 14  (endogenous variable: ADI; outcome variable: OGC and SOI) | NEQ | 0.159 | 0.027 |
|  | ACS | 0.399 | 0.000 |
|  | SRP | 0.226 | 0.002 |
|  | SAT | 0.157 | 0.018 |
|  | OGC | -0.058 | 0.840 |
|  | SOI | 0.581 | 0.000 |
|  | KAP | 0.160 | 0.008 |
|  | Gender | -0.157 | 0.057 |
|  | Age | 0.074 | 0.401 |
|  | Education | -0.050 | 0.362 |
|  | Income | -0.024 | 0.678 |
|  | ^GC^OGC | 0.220 | 0.457 |
|  | ^GC^SOI | -0.061 | 0.616 |
| Gaussian copulas of model 15  (endogenous variable: ADI; outcome variable: OGC and KAP) | NEQ | 0.159 | 0.027 |
|  | ACS | 0.399 | 0.000 |
|  | SRP | 0.226 | 0.002 |
|  | SAT | 0.157 | 0.018 |
|  | OGC | -0.054 | 0.853 |
|  | SOI | 0.511 | 0.000 |
|  | KAP | 0.328 | 0.104 |
|  | Gender | -0.156 | 0.059 |
|  | Age | 0.073 | 0.411 |
|  | Education | -0.045 | 0.417 |
|  | Income | -0.029 | 0.607 |
|  | ^GC^OGC | 0.216 | 0.467 |
|  | ^GC^KAP | -0.155 | 0.409 |
| Gaussian copulas of model 16  (endogenous variable: ADI; outcome variable: SOI and KAP) | NEQ | 0.159 | 0.027 |
|  | ACS | 0.399 | 0.000 |
|  | SRP | 0.226 | 0.002 |
|  | SAT | 0.160 | 0.017 |
|  | OGC | 0.157 | 0.000 |
|  | SOI | 0.525 | 0.002 |
|  | KAP | 0.312 | 0.187 |
|  | Gender | -0.155 | 0.059 |
|  | Age | 0.077 | 0.377 |
|  | Education | -0.048 | 0.391 |
|  | Income | -0.027 | 0.624 |
|  | ^GC^SOI | -0.011 | 0.938 |
|  | ^GC^KAP | -0.142 | 0.511 |
| Gaussian copulas of model 17  (endogenous variable: ADI; outcome variable: SAT, OGC, and SOI) | NEQ | 0.159 | 0.027 |
|  | ACS | 0.399 | 0.000 |
|  | SRP | 0.226 | 0.002 |
|  | SAT | 0.244 | 0.389 |
|  | OGC | -0.067 | 0.822 |
|  | SOI | 0.566 | 0.000 |
|  | KAP | 0.157 | 0.012 |
|  | Gender | -0.158 | 0.057 |
|  | Age | 0.075 | 0.387 |
|  | Education | -0.053 | 0.337 |
|  | Income | -0.023 | 0.695 |
|  | ^GC^SAT | -0.079 | 0.729 |
|  | ^GC^OGC | 0.227 | 0.452 |
|  | ^GC^SOI | -0.047 | 0.718 |
| Gaussian copulas of model 18  (endogenous variable: ADI; outcome variable: SAT, OGC, and KAP) | NEQ | 0.159 | 0.027 |
|  | ACS | 0.399 | 0.000 |
|  | SRP | 0.226 | 0.002 |
|  | SAT | 0.205 | 0.477 |
|  | OGC | -0.059 | 0.843 |
|  | SOI | 0.512 | 0.000 |
|  | KAP | 0.308 | 0.170 |
|  | Gender | -0.156 | 0.060 |
|  | Age | 0.074 | 0.401 |
|  | Education | -0.047 | 0.397 |
|  | Income | -0.027 | 0.631 |
|  | ^GC^SAT | -0.043 | 0.853 |
|  | ^GC^OGC | 0.220 | 0.467 |
|  | ^GC^KAP | -0.137 | 0.490 |
| Gaussian copulas of model 19  (endogenous variable: ADI; outcome variable: SAT, SOI, and KAP) | NEQ | 0.159 | 0.027 |
|  | ACS | 0.399 | 0.000 |
|  | SRP | 0.226 | 0.002 |
|  | SAT | 0.194 | 0.506 |
|  | OGC | 0.157 | 0.000 |
|  | SOI | 0.523 | 0.002 |
|  | KAP | 0.300 | 0.233 |
|  | Gender | -0.156 | 0.061 |
|  | Age | 0.078 | 0.370 |
|  | Education | -0.049 | 0.379 |
|  | Income | -0.027 | 0.640 |
|  | ^GC^SAT | -0.031 | 0.895 |
|  | ^GC^SOI | -0.008 | 0.954 |
|  | ^GC^KAP | -0.131 | 0.556 |
| Gaussian copulas of model 20  (endogenous variable: ADI; outcome variable: OGC, SOI, and KAP) | NEQ | 0.159 | 0.027 |
|  | ACS | 0.399 | 0.000 |
|  | SRP | 0.226 | 0.002 |
|  | SAT | 0.158 | 0.018 |
|  | OGC | -0.058 | 0.843 |
|  | SOI | 0.535 | 0.001 |
|  | KAP | 0.314 | 0.183 |
|  | Gender | -0.156 | 0.059 |
|  | Age | 0.073 | 0.411 |
|  | Education | -0.046 | 0.410 |
|  | Income | -0.027 | 0.628 |
|  | ^GC^OGC | 0.220 | 0.461 |
|  | ^GC^SOI | -0.021 | 0.883 |
|  | ^GC^KAP | -0.142 | 0.510 |
| Gaussian copulas of model 21  (endogenous variable: ADI; outcome variable: SAT, OGC, SOI, and KAP) | NEQ | 0.159 | 0.027 |
|  | ACS | 0.399 | 0.000 |
|  | SRP | 0.226 | 0.002 |
|  | SAT | 0.202 | 0.492 |
|  | OGC | -0.062 | 0.835 |
|  | SOI | 0.532 | 0.002 |
|  | KAP | 0.297 | 0.233 |
|  | Gender | -0.156 | 0.061 |
|  | Age | 0.074 | 0.402 |
|  | Education | -0.048 | 0.393 |
|  | Income | -0.026 | 0.646 |
|  | ^GC^SAT | -0.040 | 0.866 |
|  | ^GC^OGC | 0.224 | 0.462 |
|  | ^GC^SOI | -0.018 | 0.904 |
|  | ^GC^KAP | -0.128 | 0.563 |
| Gaussian copulas of model 22  (endogenous variable: ADI; outcome variable: NEQ, ACS, SRP, SAT, OGC, SOI, and KAP) | NEQ | 0.019 | 0.956 |
|  | ACS | 0.353 | 0.222 |
|  | SRP | -0.211 | 0.499 |
|  | SAT | 0.202 | 0.492 |
|  | OGC | -0.062 | 0.835 |
|  | SOI | 0.532 | 0.002 |
|  | KAP | 0.297 | 0.233 |
|  | Gender | -0.156 | 0.061 |
|  | Age | 0.074 | 0.402 |
|  | Education | -0.048 | 0.393 |
|  | Income | -0.026 | 0.646 |
|  | ^GC^NEQ | 0.139 | 0.649 |
|  | ^GC^ACS | 0.024 | 0.918 |
|  | ^GC^SRP | 0.406 | 0.151 |
|  | ^GC^SAT | -0.040 | 0.866 |
|  | ^GC^OGC | 0.224 | 0.462 |
|  | ^GC^SOI | -0.018 | 0.904 |
|  | ^GC^KAP | -0.128 | 0.563 |

Note: The scales with GC as a superscript refer to endogeneity corrected variables via Gaussian copulas.
